# Impact of preoperative pelvic floor muscle function on the success of surgical treatment of pelvic organ prolapse

**DOI:** 10.1007/s00192-023-05653-8

**Published:** 2023-10-11

**Authors:** Jacek Krzysztof Szymański, Małgorzata Starzec-Proserpio, Dorota Bartosińska-Raczkiewicz, Agata Krawczyk, Piotr Kukulski, Grzegorz Jakiel

**Affiliations:** 1 1st Department of Obstetrics and Gynecology, Centre of Postgraduate Medical Education, Żelazna 90 Str., 01-004 Warsaw, Poland; 2 Department of Midwifery, Centre of Postgraduate Medical Education, Warsaw, Poland; 3 Department of Medical Statistics, Centre of Postgraduate Medical Education, School of Public Health, Warsaw, Poland; 4 Department of Rehabilitation, Medical University of Warsaw, Warsaw, Poland

**Keywords:** Pelvic organ prolapse, Pelvic floor muscle function, Prolapse surgery

## Abstract

**Introduction and hypothesis:**

The objective of this study was to identify the potential characteristics of pelvic floor muscles (PFM) in the preoperative assessment that could be associated with post-surgical prolapse severity. We hypothesized that the same variables, if identified, could be addressed in preoperative rehabilitation to improve surgical results.

**Methods:**

This was a single-center prospective observational study that included women who underwent surgical pelvic organ prolapse repair between 2020–2022. Genital prolapse was evaluated according to the Pelvic Organ Prolapse Quantification (POP-Q) system. All the participants underwent a PFM assessment, including a vaginal digital assessment and manometry (Peritron™ 9300 V) before surgery and at 1-, 3-, and 6-month follow-ups. Several PFM variables were recorded: vaginal resting pressure, vaginal pressure during maximal voluntary contraction (MVC), area under the curve during a 10-second MVC, ability to correctly contract the PFMs, and reflexive activation during cough and relaxation. The primary endpoint of the analysis was objective surgical success defined as POP-Q 0 or 1 at the 6-month follow-up. Additionally, a change in pelvic floor muscle function was recorded during postoperative visits.

**Results:**

A total of 106 females were included in the study. Fifty-one were lost during the 6-month follow-up, which is a major limitation of the study. None of the examined parameters evaluating PFM were associated with surgical success. No statistically significant difference was found in MVC and PFM endurance before and after surgery. Post-surgery, a significant change was observed in the vaginal resting pressure and the ability to correct PFM activation and relaxation.

**Conclusions:**

Preoperative PFM function is not associated with surgical success 6 months after surgery.

## Introduction

It is estimated that pelvic organ prolapse (POP) may affect up to 50% of women [1] and at any stage can deteriorate their quality of life [2, 3]. The treatment options include observations in asymptomatic patients, pelvic floor muscle (PFM) training, pessary use, or a variety of surgical procedures [4]. Complications after surgical treatment are reported in a wide range (2.5–40%) of patients, depending on the surgical technique, follow-up time, comorbidities, and various definitions of the complication, while recurrences requiring subsequent surgical procedures occur in 1.4–4.4% of patients [5, 6]. Hence, the necessity to conduct research focused on optimizing the treatment of POP. Such research should be aimed at identifying areas where a possible improvement would result in lasting clinical success. Until now, several factors have been investigated. A systematic review from 2015 revealed parity, vaginal delivery, age, and body mass index (BMI) as the key risk factors regarding primary POP. With regard to POP recurrence, only preoperative stage Pelvic Organ Prolapse Quantification (POP-Q) 3 or 4 and levator ani defects were identified as risk factors [7]. Jakus-Waldman and colleagues (2020) in the secondary analysis of the OPTIMAL study identified Hispanic ethnicity, larger preoperative perineal body, and higher pretreatment Pelvic Organ Prolapse Distress Inventory scores as the risk factors for surgical failure up to 5 years after vaginal prolapse repair [8].

As PFM play an important role in the development and maintenance of pelvic floor dysfunctions [9], PFM function may be a variable worth considering in terms of predicting POP surgery success. Previous studies showed that women with POP have poorer PFM function when compared to women without POP [10–12]. It was also shown that PFM function (strength) is related to POP symptom severity [13]. The relation between PFM function and POP may reinforce the belief that preoperative PFM function could be one of the predictors of postoperative outcomes. Knowledge about specific PFM function variables and their relationship with surgical outcomes could potentially indicate factors that could be addressed in preoperative rehabilitation, possibly leading to improvements in surgical outcomes. To our knowledge, there is a scarcity of studies evaluating preoperative PFM function in women with POP in terms of surgical success [14–17].

Therefore, the main aim of this study was to investigate whether preoperative PFM function can be an independent factor of surgical success at 6-month follow-up. In addition, we wanted to track the potential change in PFM function at selected time points: after 1-, 3-, and 6-months following surgery.

## Material and methods

This prospective observational study was carried out from October 2019 to October 2022 in the First Department of Obstetrics and Gynecology, Centre of Postgraduate Medical Education, St Sophia Hospital in Warsaw, Poland. The study was approved by the institutional ethics committee (No.: 62/PB/2019), registered in the ClinicalTrials.gov database (reg. Nr. NCT04029168), and written informed consents were obtained from all the participants before they were enrolled in the study. The registered study design also includes an assessment of other outcomes, such as quality of life assessment and urinary incontinence. These issues will be the subject of subsequent publications. The STROBE checklist was followed to ensure proper reporting of the study [18].

Women admitted to surgery due to POP were included in this study. The following characteristics were recorded: age, BMI, comorbidities, number and type of deliveries, menopausal status, use of hormonal replacement therapy, and topical estrogen therapy. The exclusion criteria were previous pelvic surgery, current abnormal cervical smear or known malignancy, and mental illness precluding participation in the study. Genital prolapse was graded according to the POP-Q system [19]. The scale ranges from 0–4, where 0 means no pelvic organ prolapse, and 4 means complete prolapse. The examination was conducted by two gynecologists with 30 years of experience. Before the evaluation, the patient was asked to empty her bladder. Then, the examination was performed in the lithotomy position with bearing down [20].

The prolapse of the anterior vaginal wall, the posterior vaginal wall, and the apical defect were separately assessed. To simplify the final analysis, for the purposes of the study, the term POP-Q max was introduced to define the lowest point of genital prolapse without differentiating which part of the vagina it concerns.

### Pelvic floor assessment

All patients underwent a PFM evaluation. Examinations were performed by three experienced pelvic health physiotherapists who had completed advanced training in urogynecology and were certified by the Polish Urogynecological Society to examine pelvic floor muscles. All physiotherapists attended a mandatory half-day training together, reviewing the organizational aspects of the study and the clinical examination procedures. The training aimed to promote consistency throughout the data collection process.

The PFM assessment was performed using vaginal manometry (Peritron™ 9300 V, Laborie, Canada). It consisted of an air-filled silicone rubber sensor connected to a measuring unit via a plastic tube. The unit measured pressure in cmH_2_O. The sensor was covered with a sterile latex sleeve for each patient, and a small amount of water-soluble lubricant was applied. During the examination, three values were recorded: (1) vaginal resting pressure (as an approximated value of PFM tone), (2) vaginal pressure during maximal voluntary contraction (MVC, as a measure of PFM strength), and (3) area under the curve during a 10 s MVC (as an approximated value of PFM endurance).

Before the examination, all the participants were asked to empty their bladder to ensure their comfort during the assessment and to avoid any potential influence of bladder fullness on PFM function. They were then asked to lie down, with their knees bent. Digital palpation was always the first examination carried out to confirm the woman’s ability to contract the PFM. The following were recorded: relaxation (correct, partial/delayed, or absent), and ability to perform a correct, voluntary contraction of the PFM (isolated, voluntary PFM contraction without breath holding was considered correct). In case of incorrect voluntary PFM contraction, a short instruction was provided to secure valid manometric measurements. Additionally, we attempted an assessment of involuntary PFM contraction, evaluating perineal movement with rapid increase in intra-abdominal pressure – coughing. The women were not instructed to contract PFMs before coughing, to differentiate from the voluntary pre-contraction response presence of concurrent, reflexive perineal elevation was recorded (present or absent) [20].

After ensuring proper voluntary PFM contraction, the vaginal sensor was inserted into the vaginal canal to the full extent of the compressible portion of the device until it was above the level of the hymenal ring. The baseline vaginal resting pressure was then recorded. The value was read after about 30 s. This was done to avoid measurement bias due to the change in muscle length and possible reflexive increase of resting pressure upon probe insertion. The baseline value of vaginal resting pressure varies between individuals and may influence further measurements. To compensate for this variability and to create comparable readings between the participants, the sensor was then inflated to a preset value of 100 cmH_2_O before measuring the MVC, as suggested by the manufacturer. Following the inflation, the device was reset to zero and the vaginal pressure during MVC was measured. The women were asked to contract their PFM in and up as strongly as possible for 3 s, three times. We used an interval of 10 s between contractions. The device was reset to zero after each contraction. The mean of the three trials was recorded. After a 30-s break (to avoid muscle fatigue), the ability to sustain near-maximal or maximal contraction was assessed. It was quantified as the area under the curve for 10 s and was measured on one attempt. This value was previously used as a quantification of muscular endurance in previous studies [21, 22]. Valid measurements were ensured by the simultaneous observation of the inward movement of the perineum. Any contractions for which a retroversion of the hip or a Valsalva maneuver was noticed were discounted.

Manometry is considered a valid and reliable measure of PFM function. The vaginal squeeze pressure during PFM contraction has shown excellent test–retest (ICC = 0.88–0.96) [23] and intrarater reliability (r = 0.88) [24] for the assessment of muscle strength and endurance. Additionally, pressure measurements correlate with vaginal palpation (r = 0.646) [25] and transabdominal ultrasound (r = 0.72) [26].

Urogynecological surgical procedures were performed by two experienced gynecological surgeons. The scope of techniques included: anterior vaginal reconstruction and/or colpoperineorrhaphy, the Manchester-Fothergill procedure, six-point anterior vaginal mesh implantation, midurethral sling (transobturator and retropubic – only as an additional procedure to prolapse surgery), sacrospinous ligament fixation, and laparoscopic sacro-utero- or colpopexy.

A postoperative follow-up was carried out 1, 3, and 6 months after surgery. The anatomical outcomes of the pelvic floor’s static were assessed according to the POP-Q scale. POP-Q 0 or 1 (where 0 means no prolapse and 1 means that the point of maximum prolapse is > 1 cm above the hymen) at 6-month follow-up were considered an objective optimal surgical outcome and were the primary endpoints for the analysis. The PFM function was evaluated according to the preoperative protocol. Additionally, during the follow-up visits, patients received individualized lifestyle and PFM training instructions (3 series of 6–12 voluntary contractions sustained for 4–10 s and performed 3–4 times a week).

## Statistical analysis

The data were analyzed using STATISTICA 13 software, Statsoft, Krakow, Poland.

Absolute numbers (n) and percentages (%) of the occurrence of categories were estimated for categorical variables. Minimum and maximum values as well as arithmetic means (M) and standard deviations (SD) were estimated for numerical variables.

In the PFM assessment, three continuous variables were analyzed: (1) vaginal resting pressure; (2) strength: vaginal squeeze pressure; and (3) endurance: area under the curve. Three categorical variables were also investigated: (1) perineal elevation with cough (as a proxy to reflexive, involuntary PFM contraction); (2) correct voluntary contraction of PFMs; and (3) PFM relaxation.

To compare patients’ characteristics and PFM assessment between surgical success and failure at 6 months, the following statistical tests were used:

- U Mann–Whitney test for numerical variables
- Fisher’s exact test for categorical variables.

In the analyses of the POP-Q scale, first the absolute numbers (n) and percentages (%) for POP-Q = 0, 1, 2, 3 and 4, respectively, were estimated, followed by arithmetic mean (M) and standard deviation (SD). The Wilcoxon signed rank test was used to compare POP-Q grading between each pair out of four time points: baseline, 1 month, 3 months, and 6 months after surgery.

Lastly, binary logistic regression was used to estimate the odds ratio of cured (POP-Q = 0 or 1) versus failed (POP-Q = 2, 3 or 4) at 6 months after surgery. Five variables of pelvic floor muscles at baseline were considered as predictors. PFM relaxation was omitted in logistic regression analysis because of the small counts for two out of three categories: none and partial/delayed.

Manometry could not be performed in 10 patients due to pain during attempted examination. These variables in different time points were omitted from statistical analysis. The use of statistical methods for four repeated measurements was not possible, only for comparison purposes in pairs between two time points. 

The significance level was assumed to be 0.05 in all the statistical tests.

## Results

A total of 106 females were included in the study. Fifty-one were lost during the 6-month follow-up, resulting in the inclusion of 55 at the 6-month follow-up analysis. The patients who were followed up at the 1-month and 3-month but not 6-month time point were not included in the final analysis because we believe that the 6-month follow-up is the shortest period after which the success or failure of the treatment can be determined. Despite the loss of 51 participants from follow-up, the remaining 55 were sufficient to include five variables in logistic regression.

No statistically significant differences were found between the participants who attended the 6-month follow-up visit and those who were lost to follow-up, as well as between those cured and those with treatment failure in terms of age, comorbidities, menopausal status, and PFM function. Only the higher BMI was found to reach statistical significance in patients with treatment failure. The characteristics of the participants are presented in Table 1.

**Table 1:** Patients’ characteristics at baseline

Variable, parameter	Total sample (N = 106)	Surgical success of POP treatment (SPOP-Q value 0 or 1 at 6 months after surgery) (N = 38)	Surgical failure of POP treatment (SPOP-Q value 2 or 3 or 4 at 6 months after surgery) N = 17)	p
Age (years), M ± SD	57.7 ± 12.8	59.1 ± 11.9	64.1 ± 11.3	0.137
BMI (kg/m^2^), M ± SD	27.3 ± 4.4	26.9 ± 4.5	29.9 ± 3.4	0.011
Diabetes, n (%)	7 (6.6)	1 (2.6)	0 (0)	1
Hypertension, n (%)	32 (30.2)	12 (31.6)	8 (47.1)	0.27
Hyporhyroidism, n (%)	13 (12.2)	5 (13.2)	1 (5.9)	0.654
Hyperthyroidism, n (%)	4 (3.8)	2 (5.3)	0 (0)	1
Postmenopausal, n (%)	72 (67.9)	28 (73.9)	14 (82.4)	0.37
HRT, n (%)	5 (4.7)	2 (5.3)	0 (0)	1
Topical estrogens, n (%)	18 (17.0)	6 (15.8)	3 (17.7)	1
Parity, M ± SD	2.3 ± 1.4	2.2 ± 0.6	2.1 ± 1.2	0.243
Vaginal resting pressure (cmH_2_O), M ± SD	25.4 ± 6.9	24.2 ± 6	26.9 ± 7.4	0.592
Strength: Vaginal squeeze pressure (cmH_2_O), M ± SD	15.2 ± 10	14.1 ± 8.2	16.7 ± 12.7	0.715
Endurance: Area under the curve, M ± SD	1072 ± 771	1051 ± 809	964 ± 673	0.928
Reflexive activation of PFM, yes, n (%)	25 (25)	6 (15.8)	3 (17.7)	0.574
Correct activation of PFM, yes, n (%)	33 (33)	13 (34.21)	3 (17.7)	0.336
PFM Relaxation:				
yes, n (%)	77 (78.57)	30 (78.95)	13 (81.25)	0.673
partial/delayed, n (%)	12 (12.24)	5 (13.16)	1 (6.25)	
no, n (%)	9 (9.18)	3 (7.89)	2 (12.5)	

no data for one patient

From the examined parameters evaluating the preoperative function of the PFM, none of them was associated with the maximal value of the POP-Q scale and the surgical success (defined as POP-Q 0 or 1) at 6-month follow-up (Table 2).

**Table 2:** Predictive factors of surgical success of POP treatment (SPOP-Q value 0 or 1). Logistic regression model for cured (ref = failed) at 6 months after surgery compared to baseline

Predictor	OR (95% CI)	p
Vaginal resting pressure (cmH_2_O), at baseline	0.938 (0.853–1.033)	0.193
Strength: Vaginal squeeze pressure (cmH_2_O), at baseline	0.972 (0.912–1.036)	0.389
Endurance: Area under the curve, at baseline	1 (0.999–1.001)	0.794
Reflexive activation of PFM (ref = no), at baseline	0.875 (0.191–4.007)	0.863
Correct activation of PFM (ref = no), at baseline	2.427 (0.589–10))	0.22

OR, odds ratio

**Fig. 1:**
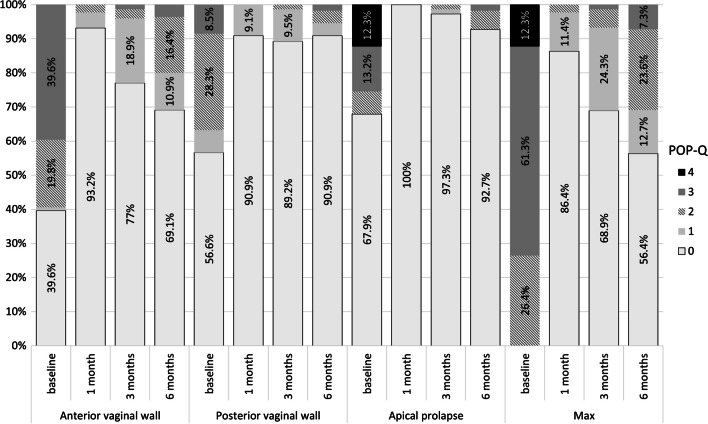
Changes in genital prolapse in POP-Q scale (%)

**Fig. 2:**
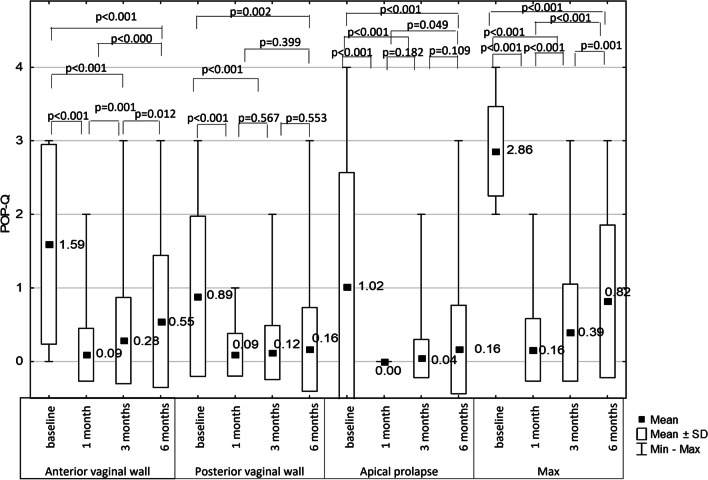
Changes in genital prolapse in POP-Q scale (median)

Of the 55 patients who remained in the 6-month follow-up, 69.09% were cured (0–1 according to the POP-Q scale. For the anterior vaginal wall, a high cure rate of over 95% gradually decreased to 80% at the 6-month follow-up. In contrast, for the posterior vaginal wall and apical defect, the high cure rates observed at 1 month remained relatively constant over 6 months. Therefore, the decrease in the effectiveness of treatment observed in POP-Q max from approximately 95% after 1 month to 69% after 6 months is most likely related to the deterioration of the statics of the anterior vaginal wall (Figs. 1 and 2).

## Discussion

The design of this study provided a unique opportunity to investigate the association of PFM function with surgical outcomes measured in women with POP. The study aimed to investigate whether certain parameters related to preoperative PFM function could be predictors of surgical success defined as POP-Q 0 or 1 at 6-month follow-up. We did not find any associations between preoperative PFM function and surgical success.

In a study from 2017, performed by Schachar and colleagues, weak PFM have been associated with a higher risk of recurrence and surgical failure, defined as point Ba of 0 or greater on the POP-Q scale [15]. Based on the PFM strength assessment on palpation (Modified Oxford Scale), patients with an inability to contract PFM demonstrated a significantly higher risk of surgical failure than those with weak and strong muscles (13.89% vs. 3.80%). This study, contrary to ours, was retrospective, but with a longer follow-up period and a higher number of patients under observation. In our study, apart from elements of the Oxford scale, vaginal manometry was also used for a more detailed analysis of the individual parameters that make up the assessment of the pelvic floor and to improve interrater reliability. None of the assessed parameters turned out to be a predictor of therapeutic success. It is possible that a different method of analysis, identifying groups with strong and weak pelvic floor muscles based on the analyzed parameters, would bring different results. Probably, however, it would be a repetition of those received by Schachar and colleagues [15]. Furthermore, categorizing muscle strength would undermine the power of our study. Therefore, in the analysis, we treated muscle strength as a continuous variable. Another retrospective analysis including 358 women post prolapse and/or incontinence surgery with a 5-month follow-up period found a correlation between the strength of the levator ani contraction and subsequent surgery for POP, and between a widened genital hiatus and recurrent prolapse. In our study, we focused on the assessment of variables related to PFM function [14]. The impact of the preoperative assessment of prolapse in the POP-Q scale on the outcome of treatment (where GH would certainly be included) was not assessed. Therefore, we did not assess genital hiatus as it is part of the POP-Q scale and not the assessment of PFM function. Interesting conclusions can be drawn from the work of Nyhus and colleagues [16], who assessed the impact of levator ani trauma on the recurrence of prolapse. An association between a levator ani avulsion and surgical failure has been demonstrated based on ultrasound imaging. Interestingly, absent or weak PFM contraction was associated with a reduced risk of bulge sensation after surgery. According to the authors, an explanation for this phenomenon may be the more frequent reporting of POP symptoms by younger women, who are more likely to present with normal and strong contractions. In addition, neurological damage to the pelvic floor can affect both the strength of muscle contraction and the bulge sensation. The essence of our study was the clinical aspect to be used in everyday practice. For this reason, we did not use ultrasonography since the diagnosis of levator ani trauma is not a routine preoperative procedure. It is possible that pelvic organ support is provided mainly by the interaction between the levator ani muscles and the connective tissue that attach the uterus and the vagina to the pelvic sidewall [27], while the PFM functional parameters (e.g., strength) are of less importance. This would be in line with the current data synthesis and recommendations presented by the 7th International Consultation on Incontinence, stating that PFM training was associated with a reduction of prolapse and PFM symptoms, yet its role in the reduction of the prolapse stage is uncertain [28]. Another factor that could explain our results is the choice of dependent variable – surgical success measured with the POP-Q scale. It is plausible that preoperative PFM function exerts an influence on the postoperative results; however, this effect is too subtle and, hence, is not detected by the POP-Q scale.

Similar to our study, several other authors reported changes in PFM function following surgery. Three trials showed an improvement in PFM strength 3 months after surgery; however, the obtained value of PFM strength did not reach the level of PFM activity of the control group without POP [29, 30]. In turn, McConnell and colleagues showed a significant increase in peak and mean MVC values (measured with an intra-vaginal pressure device) in women with posterior colporrhaphy, with no such effect in the anterior repair group. A possible explanation for this phenomenon, according to the authors, is the better strengthening of the pelvic floor muscles with posterior repair [31]. In another study, Duarte and colleagues reported no difference in the maximal value of MVC before and 40 days after surgery, although the authors highlight the improvement in PFM average contraction after POP surgery [32]. It is possible that the restoration of the correct anatomy by surgery improves the function of the pelvic floor. A randomized controlled trial from 2020 on 159 patients showed an improvement in pelvic floor function as a result of surgical treatment [33]. Improvement in muscle contraction assessed by palpation on the Modified Oxford Scale and in ultrasound assessment of the levator hiatal anteroposterior diameter was reported in all patients after surgery. The authors found no additional benefit from preoperative PFM training on PFM strength, POP symptoms, and postoperative symptom recurrence. However, according to the authors, these women could not have performed better because they had already undergone a presurgery examination and had received preoperative instructions. Contrary to these findings, our study did not find a significant improvement in average MVC and PFM endurance postoperatively compared to the initial assessment. However, we noted a statistically significant improvement in vaginal resting pressure and involuntary muscle contraction bordering on statistical significance. Previous studies, although assessing vaginal resting pressure, did not analyze this parameter in relation to surgical outcomes, focusing mainly on MVC. In our opinion, the vaginal resting pressure (as a proxy measure to PFM tone) may be an important parameter contributing to the stabilization of the pelvic floor during daily activity in the absence of rapid increases in intra-abdominal pressure by affecting the closure of the levator hiatus [34]. It was also found to be significantly lower in women with POP, especially in combination with weak PFM [10]. Thus, the improvement of this variable could be a benefit of reconstructive surgery. On the other hand, an improvement in involuntary muscle contraction during cough enhances the pelvic floor’s response to a sudden increase in intra-abdominal pressure [35]. However, we cannot be sure of the conclusion that the changes obtained can only be attributed to the surgical procedure. During postoperative visits after 1, 3, and 6 months, our patients received individualized recommendations regarding lifestyle and PFM training, which could influence the assessed PFM function. Our results are in line with the systematic review based on 21 studies which found no effect of POP surgery on improving pelvic floor strength and function in the short to medium follow-up [36]. The authors emphasize that the low quality and heterogeneity of the researched papers made it impossible to draw certain conclusions. Moreover, none of the studies examined the relationship between PFM measures and POP quantification before and after surgery.

In our study, we achieved an objective cure and improvement in over 92% of cases. In terms of the examined variables, the characteristics of the failure patients did not differ from those of cured patients. However, due to their small number, the analysis of the characteristics of this group does not allow conclusions to be drawn. In 15% of patients, we noticed a gradual deterioration of the anterior vaginal wall within 6 months compared to 1 month after surgery, while the achieved stabilization of the posterior vaginal wall was maintained. In the observations of other authors, no differences in postoperative statics between the anterior and posterior vaginal walls were observed. However, these studies were characterized by a longer follow-up and were dedicated to a specific surgical technique [37, 38]. Our results should be interpreted with great caution as they refer to a variety of surgical procedures and the small sample size made it impossible to perform a sub-analysis for each of the procedures. Moreover, such an analysis was not the purpose of this study.

The lack of observed relationship between the function of the PFM and the surgical success resulting from our study should not lead to the exclusion of the PFM assessment from the qualification process for surgical treatment. We believe that such assessment should be included in routine preoperative management. Moreover, there is a need for further studies on larger groups of patients with a longer follow-up period, as POP surgical treatment assumes obtaining a long-term beneficial effect. To measure more parameters describing the PFM, it will also be beneficial to implement more accurate, validated devices.

The aim of the authors was the practical aspect of the study, which could be used in everyday clinical practice. Therefore, patients with different POP stages were included in the study, which, with many assessed parameters, increased the clinical reliability of the study and constitutes the strength of this research. Another strength of this study is the use of validated methods, such as manometry, to evaluate PFM function. We are aware that manometry is not routinely used in the preoperative assessment of women with POP. However, for the purposes of the study, we wanted to assess pelvic floor muscle function more precisely. Areas of PFMs potentially identified as requiring training could, in routine, only be practiced with palpation, particularly that, as mentioned above, manometry is correlated with vaginal palpation and transabdominal ultrasound [25, 26]. In addition, the variety of surgical procedures used increases the generalizability of our findings.

The limitation of the study is the relatively short, only 6-month follow-up and the significant loss of patients during this period. The loss of 48% of the participants from observation significantly reduces the power of the analysis. The reasons for such a large loss are certainly varied. It can be assumed that some of the women satisfied with the effect of the surgery did not see the need for further follow-ups. Especially during the COVID-19 pandemic, patients who felt cured may have been afraid of the risks associated with visiting a hospital. In contrast, patients who failed could have lost confidence and moved on to treatment at other centers. The reason may also result from the mixed model of healthcare in Poland. Some of the participants were able to return to care in private practices. On the other hand, patients from distant places of residence could come to the hospital for surgical treatment only and remain under medical care in their area. Finally, unforeseen fortuitous events could prevent a certain number of patients from coming for a follow-up visit. We are aware that such a large loss of patients may result in significant bias, and completely different outcomes could be obtained when analyzing a larger group of patients. Therefore, the obtained results should be interpreted with caution. Another limitation of this study is the assessment of involuntary, reflexive PFM contraction during coughing. Although we followed the ICS terminology report [20] and did not instruct women to contract PFMs, we cannot be certain that the observed perineal elevation was not a voluntary (learned) response. An additional limitation may be the lack of subjective assessment of the treatment results using questionnaires assessing quality of life. We deliberately did not include this analysis because we focused on the objective assessment of both the function of the pelvic floor muscles before surgery and the results of the surgical treatment. All the patients completed appropriate questionnaires assessing quality of life related to pelvic floor dysfunction. The analysis of these materials will be the subject of the next article.

## Conclusions

The study did not show a clear association between the preoperative PFM function and the success of surgical treatment assessed with the POP-Q grading system. However, the clinical implications of this result are not unambiguous and require additional research. It seems that the complexity of pelvic floor pathology makes it difficult to identify a single factor that would be responsible for improving the effectiveness of treatment. Therefore, further extensive research is needed to understand the complex pathophysiology of the pelvic floor to achieve long-lasting therapeutic success.

## Data Availability

All data are available from the authors.
